# Short- and Long-Term Regulation of HuD: A Molecular Switch Mediated by Folic Acid?

**DOI:** 10.3390/ijms241512201

**Published:** 2023-07-30

**Authors:** Nicoletta Marchesi, Pasquale Linciano, Lucrezia Irene Maria Campagnoli, Foroogh Fahmideh, Daniela Rossi, Giosuè Costa, Francesca Alessandra Ambrosio, Annalisa Barbieri, Simona Collina, Alessia Pascale

**Affiliations:** 1 Department of Drug Sciences, Pharmacology Section, University of Pavia, 27100 Pavia, Italy; lucreziairenem.campagnoli01@universitadipavia.it (L.I.M.C.); foroogh.fahmidehtavako01@universitadipavia.it (F.F.); annalisa.barbieri@unipv.it (A.B.); 2 Department of Drug Sciences, Medicinal Chemistry Section, University of Pavia, 27100 Pavia, Italy; pasquale.linciano@unipv.it (P.L.); daniela.rossi@unipv.it (D.R.); simona.collina@unipv.it (S.C.); 3 Department of Experimental and Clinical Medicine, University “Magna Græcia” of Catanzaro, Campus “S. Venuta”, 88100 Catanzaro, Italy; gcosta@unicz.it (G.C.); ambrosio@unicz.it (F.A.A.); 4 Net4Science Academic Spin-Off, University “Magna Græcia” of Catanzaro, 88100 Catanzaro, Italy; 5 Associazione CRISEA-Centro di Ricerca e Servizi Avanzati per l’Innovazione Rurale, 88055 Catanzaro, Italy

**Keywords:** ELAV/HuD, BDNF, folic acid, neurodegenerative diseases, Alzheimer’s disease

## Abstract

The RNA-binding protein HuD has been shown to play a crucial role in gene regulation in the nervous system and is involved in various neurological and psychiatric diseases. In this study, through the creation of an interaction network on HuD and its potential targets, we identified a strong association between HuD and several diseases of the nervous system. Specifically, we focused on the relationship between HuD and the brain-derived neurotrophic factor (BDNF), whose protein is implicated in several neuronal diseases and is involved in the regulation of neuronal development, survival, and function. To better investigate this relationship and given that we previously demonstrated that folic acid (FA) is able to directly bind HuD itself, we performed in vitro experiments in neuron-like human SH-SY5Y cells in the presence of FA, also known to be a pivotal environmental factor influencing the nervous system development. Our findings show that FA exposure results in a significant increase in both HuD and BDNF transcripts and proteins after 2 and 4 h of treatment, respectively. Similar data were obtained after 2 h of FA incubation followed by 2 h of washout. This increase was no longer detected upon 24 h of FA exposure, probably due to a signaling shutdown mechanism. Indeed, we observed that following 24 h of FA exposure HuD is methylated. These findings indicate that FA regulates BDNF expression via HuD and suggest that FA can behave as an epigenetic modulator of HuD in the nervous system acting via short- and long-term mechanisms. Finally, the present results also highlight the potential of BDNF as a therapeutic target for specific neurological and psychiatric diseases.

## 1. Introduction

Neurodegenerative diseases (NDs) are a heterogeneous group of diseases characterized by a progressive loss of function of the autonomic, peripheral, or central nervous system (CNS). These include Alzheimer’s disease (AD), Parkinson’s disease, multiple sclerosis, amyotrophic lateral sclerosis, and other neurological disorders. The WHO ranked NDs at 7th place in the leading causes of death worldwide. Moreover, the health, economic, and social burden caused by NDs is expected to dramatically rise in the next few decades as a result of the global population growth and aging. Therefore, the WHO recognized NDs as a global public health problem and included them in the top four challenging diseases that medicine and pharmacology should quickly address in the near future. The current, yet decade-old, pharmacological treatments for NDs are mainly symptomatic or focused on slowing down as much as possible the progression of the disease. For instance, concerning AD, in 2021, after 20 years of research failures in this field, FDA approved aducanumab, a monoclonal antibody able to target oligomeric and fibrillary beta-amyloid (Ab) protein aggregates in the brain, hence interrupting amyloid aggregation kinetics. However, reports of serious complications potentially related to the treatment have cast a shadow on that decision. Thus, research on new strategies, targets, and, consequently, new drugs to prevent neurodegeneration or to act as neurodegenerative disease-modifying therapies is still demanding [1]. Among the new putative therapeutic targets, RNA-binding proteins (RBPs), including the ELAV family, have gained attention. RBPs play a prominent role in modulating various aspects of RNA metabolism, including splicing, polyadenylation, nucleo-cytoplasmic shuttling, intracellular localization, stability, and translation of target mRNAs, thus contributing to a dynamic regulation of gene expression. This RBPs-mediated regulation has a strong impact on the levels of proteins that control key cellular functions such as proliferation, development, differentiation, and death. Indeed, a tight regulation of the expression of proteins involved in these biological processes is critical, and its dysregulation is linked to the pathogenesis of several diseases [2,3,4,5,6].

The ELAV family encompasses four members, namely HuR (ELAVL1), HuB (ELAVL2), HuC (ELAVL3), and HuD (ELAVL4) [7,8]. HuR is ubiquitously expressed, and it is mainly implicated in cell growth and cell cycle regulation [6]. Conversely, HuB, HuC, and HuD are the neuron-specific members of the family (nELAV), since they are mainly expressed in the nervous system, and they regulate the fate of target mRNAs coding for pivotal proteins taking part in key functions of neuronal cells. Among all the neuronal RBPs, HuD has been intensively investigated. A number of studies have leveraged the combination of mRNA-RBP complex purification methods and bioinformatic analyses, striving to identify the cellular targets regulated by HuD and thereby elucidating its biological function [9,10]. The gene ontology and the computational biological pathway analyses of identified HuD mRNA targets have unveiled the implication of this RBP in regulating neuronal differentiation and development, nerve regeneration, cellular response towards oxidative stress, repairing of the nervous tissue after nerve injury, as well as synaptic plasticity and memory processes [11,12,13,14,15,16,17]. Interestingly, recent research revealed that HuD may also indirectly regulate mRNA levels by controlling the expression of non-coding RNAs such as circular RNAs (circRNAs) and microRNAs (miRNAs). Indeed, deletion of HuD in the striatum has been observed to alter the levels of circRNAs and miRNAs and to affect their interactions with mRNAs. Bioinformatic analyses detected HuD-binding adenine/uracil-rich elements (ARE) in approximately 26% of brain-expressed circRNAs, indicating that HuD interactions with circRNAs might regulate their expression and transport. The subsequent alterations in HuD-regulated circRNA networks could thus influence neuronal differentiation and synaptic plasticity. Further bioinformatic examination of differentially expressed miRNAs revealed that the most likely mRNA targets of these miRNAs are associated with biological pathways and networks linked to transcriptional and epigenetic regulators (i.e., Jun, EGR3, MECP2, HDAC 1 and 9, and SMARC2), including BDNF and its associated proteins. Interestingly, the two foremost biological networks of these targets center on two proteins associated with neurodegeneration: the amyloid precursor protein (APP), which is linked to Alzheimer’s disease, and Huntingtin (HTT), a genetic mutation associated with Huntington’s disease [18].

As a result, alterations in the expression of HuD-targeted mRNAs are associated with neurodegeneration and mood disorders, epilepsy, schizophrenia, and intellectual disabilities [15,19,20]. Moreover, alterations in HuD activity through different post-translational modifications, including phosphorylation, ubiquitination, and sumoylation, can alter the binding affinity of HuD for its target mRNAs. As an example, phosphorylation of HuD in threonine residues by PKCα has been shown to modulate the stability of its down-stream target GAP-43 mRNA and to affect nELAV/HuD localization, also favoring HuD binding with polysomes [21,22]. Also, PKCα-mediated HuD phosphorylation is able to counteract an ADAM10 deficit following Ab challenge [23]. Furthermore, methylation of HuD in arginine residues by CARM1 (coactivator-associated arginine methyltransferase 1) has been shown to reduce HuD function and the binding with its target mRNAs, leading to their decreased post-transcriptional regulation. Conversely, an increase in non-methylated, HuD favors binding with the neuronal mRNA targets, promoting differentiation and elongation of neurites, and contributing to the proper development of the nervous system [24].

Accordingly, these findings suggest HuD as a novel target for the development of brand-new pharmacological interventions aimed at counteracting NDs. In addition to the comprehensive biochemical and functional characterization of HuD, the availability of crystallographic structures for the first two RNA recognition motif (RRM) domains of the HuD protein in complex with C-FOS mRNA (PDB ID: 1FXL) and TNFα mRNA (PDB ID: 1G2E [25]), together with a combined NMR and in silico approaches, has contributed to the expansion of our understanding of the structural characteristics of the protein domains interacting with the target mRNAs and the postulation of a putative ligand-binding site [19,26,27,28,29,30]. These insights pave the way for Structure-based Drug Design.

**Figure 1:**
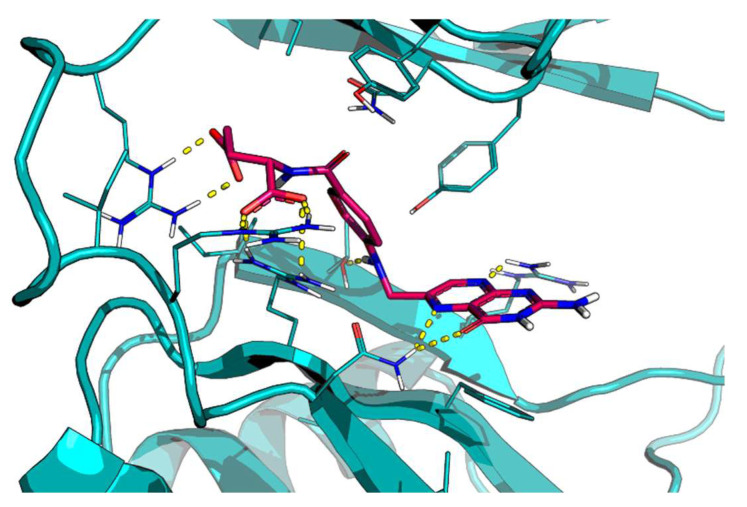
Predicted docking pose of folic acid (stick, violet carbon) at the HuD RNA-recognition motifs (RRMs)-1 and -2 binding site (teal cartoon). Important interacting residues are in the stick representation. Model atoms, except for carbons, are color-coded with oxygen (red) and nitrogen (blue). H-bonds are represented as yellow dotted lines.

Starting from these premises, we performed a drug repurposing study that allowed the identification of new ligands able to bind HuD, thus potentially influencing the formation of HuD-mRNA complexes. More in detail, by combining a virtual screening of natural compounds and FDA-approved drugs databases and STD-NMR studies, we selected folic acid (FA), rosmarinic acid, cefazolin, and enalapril maleate. Molecular dynamics studies performed to further investigate the ligand–protein interaction profile further support STD-NMR results, suggesting FA as the most attractive HuD binder (Figure 1; see also [31]).

FA, also known as vitamin B9, has fundamental roles in CNS function at all ages, and it has been recognized as a crucial environmental factor for the nervous system development. From the early fetal stages of the formation of the presumptive spinal cord and brain to the maturation and maintenance of the nervous system during infancy and childhood, as well as during the entire adult life, folate levels and their supplementation have been considered influential in the clinical outcomes of neurological diseases. Moreover, several studies proved the role of FA in the prevention of CNS developmental disorders, mood disorders, and dementias, including AD and vascular dementia. However, notwithstanding the vast epidemiological information recorded on folate function and neural tube defects, neural development, and neurodegenerative diseases, the mechanisms of folate action in the developing neural tissue is remaining still elusive.

Given the involvement of HuD in the maintenance and function of the CNS, our identification of FA as a potential ligand of HuD represented another brick in the wall to unravel the mechanism of action of FA at CNS level.

Accordingly, to gain a deeper insight into the effect of FA on HuD, in this study, we investigated at the cellular level the impact of FA on HuD expression, and we explored how this modulation influenced the regulation of specific HuD down-stream targets, crucial for maintaining a proper CNS function or for being involved in the development of NDs selected through the creation of a protein–protein interaction network.

## 2. Results

### 2.1. Protein–Protein Interaction Network of the Targets Controlled by HuD

**Figure 2:**
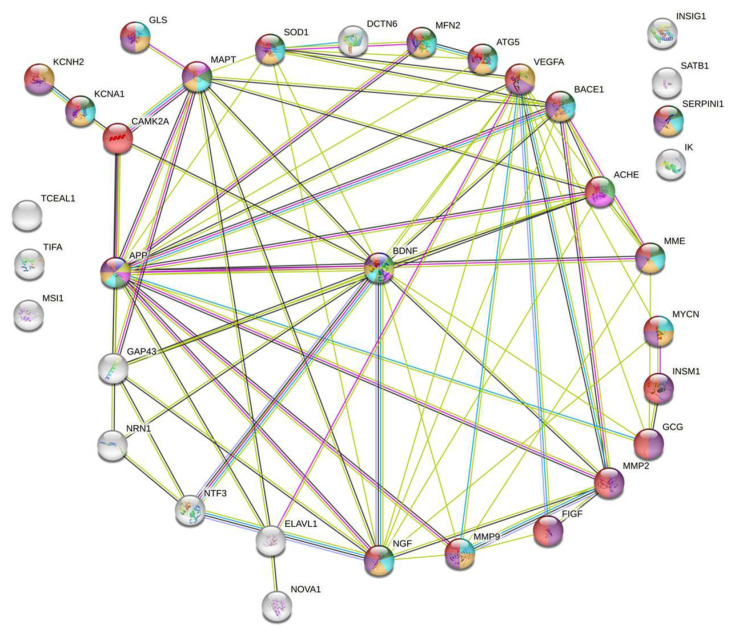
Protein–protein interaction network (PPIN) of the proteins whose mRNA is modulated by HuD and that are implicated in CNS diseases. PPIN was realized by the STRING tool. Nodes that are strongly connected are positioned closely together and have a greater number of edges entering each node. The nodes of the same color represent the same disease-gene associations with HuD. ACHE: Acetylcholinesterase, APP: Amyloid Precursor Protein, ATG5: autophagy related 5, BACE1: Beta-Secretase 1, BDNF: Brain-derived neurotrophic factor, CAMK2A: Calcium/Calmodulin-Dependent Protein Kinase II Alpha, DCTN6: Dynactin Subunit 6, SOD1: superoxide dismutase 1, ELAVL1: ELAV-like protein 1, GAP43: Growth-Associated Protein 43, GCG: glucagon, GLS: Glutaminase, IK: Ikaros, INSIG1: Insulin-Induced Gene 1, INSM1: Insulinoma-associated protein 1, KCNA1: Potassium Voltage-Gated Channel Subfamily A Member 1, KCNH2: Potassium Voltage-Gated Channel Subfamily H Member 2, MAPT: Microtubule-associated protein tau, MFN2: mitofusin 2, MME: Membrane Metallo endopeptidase, MMP2: Matrix Metallopeptidase 2, MMP9: Matrix Metallopeptidase 9, MSI1: Musashi 1, MYCN: MYCN proto-oncogene, NGF: nerve growth factor, NOVA1: ventral neuron-specific protein 1, NTF3: Neurotrophin 3, NRN1: Neuritin 1, SATB1: special AT-rich sequence-binding protein-1, SERPINI1: Serpin Family I Member 1, TCEAL1: Transcription Elongation Factor A Like 1, TIFA: TRAF Interacting Protein With Forkhead-Associated Domain, VEGFA: Vascular endothelial growth factor A.

The PPI network generated by STRING consisted of 33 nodes (Figure 2) and 76 edges. The number of edges is significantly larger than the one expected for a random network of the same size (degree ≤ 10–16); the nodes were more connected than randomly distributed. It suggested that the PPI network could be considered as a relatively small world in comparison with the random graph, and the proteins might be biologically relevant. The results of the topological analyses showed that BDNF was a hub extensively connected with their neighbors in the network (with the largest being k = 14) and the bottleneck had significant control over the network (BC = 0.151) in the PPI network. Moreover, BDNF is involved in 9 out of the 13 CNS-related pathological pathways examined (i.e., Huntington disease, amnestic disorder, toxic encephalopathy, cognitive disorder). BDNF is a neurotrophin that emerged as a key regulator of neuronal survival and differentiation, and it is strongly implicated in synaptic plasticity linked to learning and memory processes. Further, alterations in BDNF levels and signaling have been identified in various NDs and have been also associated with symptom onset and disease progression [32]. Among the 14 targets of HuD involved in neuronal and non–neuronal pathologies interconnected with BDNF, it is noteworthy to point out the interconnection with the Growth Associated Protein 43 (GAP43) [14,33,34], a primary contributor of neurite outgrowth, the Nerve Growth Factor (NGF) [35], which has an impact on neuronal differentiation, neurogenesis, dendritic maturation, neuronal plasticity, synaptic transmission, and neuronal signaling pathways, the Amyloid Precursor Protein (APP), whose abnormal processing leads to the production and accumulation of Aβ peptides, thus contributing to AD pathogenesis [36], and the Vascular Endothelial Growth Factor (VEGF), whose reduced levels or an impaired signaling may take part in NDs progression by compromising the neuroprotective mechanisms [37].

### 2.2. Effect of Folic Acid on Cell Viability and BDNF mRNA Content

The cytotoxicity of FA on human neuroblastoma SH-SY5Y cells was examined via MTT assay at different concentrations (100 nM, 1 μM, and 100 μM) and at both 24 and 48 h of exposure. The results, expressed as a percentage of cell viability, are reported in Figure S1. As expected, FA shows a good safety profile with no significant cell death vs. control at all the tested concentrations and times of exposure.

**Figure 3:**
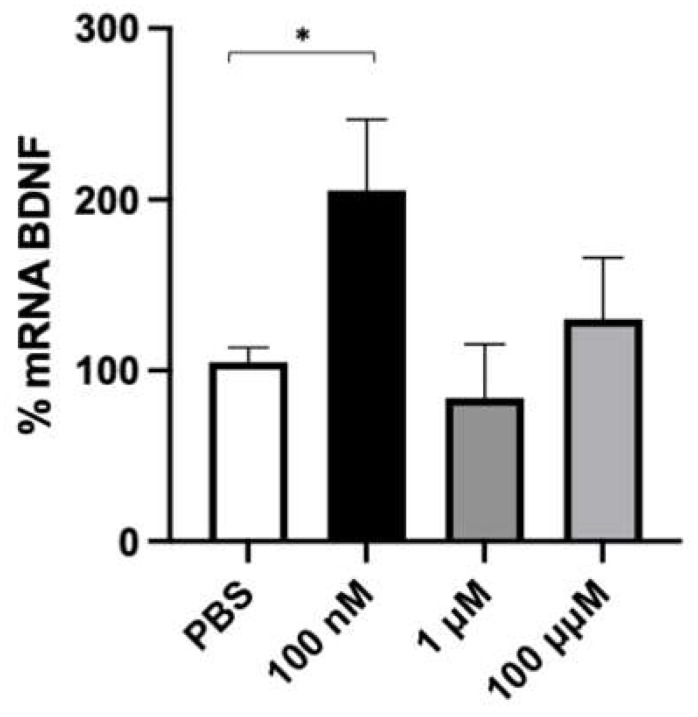
Effect of folic acid on BDNF transcript. SH-SY5Y cells were treated for 2 h with increasing concentrations of folic acid (0.1–1–100 μM) or phosphate-buffered saline (PBS) as vehicle control. mRNA levels were evaluated via RT-PCR. Each value represents the mean ± S.E.M. of independent experiments with respect to the control (100%). Statistical analysis was performed by two-way ANOVA followed by Dunnett’s Multiple Comparisons test; * *p* < 0.05 vs. control, *n* = 3 independent samples.

To identify the optimal concentration of FA to be used in the subsequent experiments, SH-SY5Y cells were treated with 100 nM, 1 μM, and 100 μM FA for 2 h. This timeframe was chosen based on previous experiments and on our own experience as being optimal for BDNF transcriptional activity, mRNA half-life (t_1/2_ 132 ± 30 min) [38], and protein expression. As reported in Figure 3, FA induced a two-fold significative increase in BDNF mRNA levels at 100 nM, whereas no variation in BDNF transcript with respect to the control was detected at higher concentrations (1 and 100 mM). This profile likely indicates a concentration-dependent effect of FA on BDNF transcript content. Based on these results, 100 nM FA was chosen as the optimal concentration for the following experiments.

### 2.3. Effect of Folic Acid on HuD and BDNF Expression

**Figure 4:**
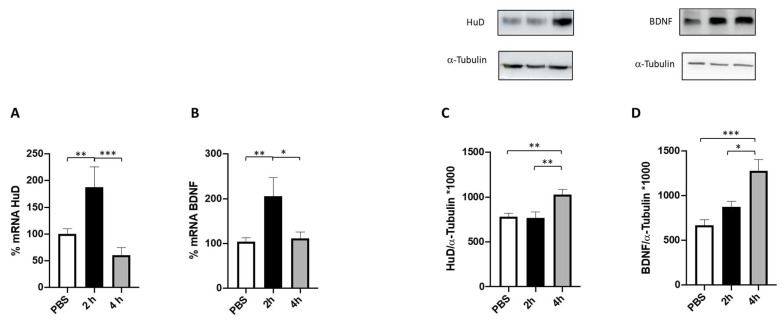
HuD and BDNF expression following 2 and 4 h of folic acid treatment. (**A**,**B**) Determination of HuD (**A**) and BDNF (**B**) mRNA levels via RT-PCR in SH-SY5Y cells exposed to solvent (phosphate-buffered saline; PBS) or to folic acid (FA) for 2 (2 h) and 4 h (4 h). The amounts of the total mRNA were normalized using the corresponding levels of GAPDH mRNA. The values are expressed as mean percentages ± S.E.M. with respect to the control (100%). * *p* < 0.05, ** *p* < 0.01, *** *p* < 0.001, Tukey’s multiple comparisons test, *n* = 3 independent samples. (**C**,**D**) Representative cropped Western blotting (upper panel) and densitometric analysis (lower panel) of HuD (**C**) and BDNF (**D**) proteins and the respective a-tubulin in the total homogenates of SH-SY5Y cells following exposure to solvent (PBS) or FA for 2 h and 4 h. Results are expressed as mean grey levels ratios (mean ± S.E.M.) of HuD/a-tubulin (**C**) and BDNF/a-tubulin (**D**) ×1000. * *p* < 0.05, ** *p* < 0.01, *** *p* < 0.001, Tukey’s multiple comparisons test, *n* = 3–7 independent samples.

The effect of 100 nM FA on both HuD and BDNF mRNA content and the relative protein expression was evaluated at two timeframes, namely 2 and 4 h. As reported in Figure 4 (panels A and B), FA was able to significatively increase BDNF and HuD mRNA levels after 2 h of treatment, whereas a return to the baseline level for both transcripts was observed after 4 h of incubation.

To further investigate whether the observed increase in HuD and BDNF mRNA levels after FA treatment translates into an effective promotion of HuD and BDNF protein expression, their protein levels were measured after 2 and 4 h of FA exposure. The obtained results show a significant increase in the amount of both proteins after 4 h of FA exposure, while no changes were observed after 2 h compared to the control (Figure 4, panels C and D). This result is in line with the time lapse between gene transcription and protein expression.

Based on these findings, we then performed a washout period after 2 h of FA treatment to understand if this time of exposure is enough to activate the FA-dependent HuD/BDNF cascade.

**Figure 5:**
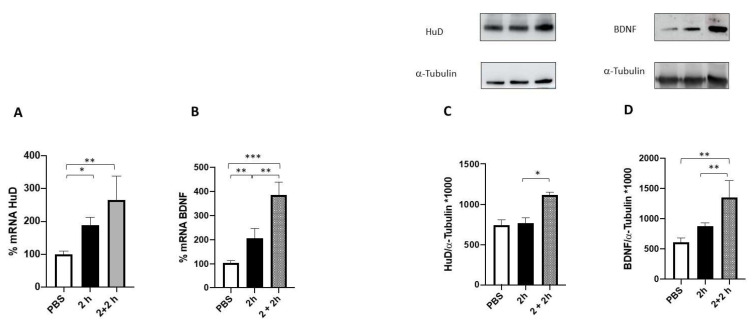
HuD and BDNF expression following 2 h + 2 h of washout of folic acid treatment. (**A**,**B**) Determination of HuD (**A**) and BDNF (**B**) mRNA levels via RT-PCR in SH-SY5Y cells exposed to solvent (phosphate-buffered saline; PBS) or to folic acid (FA) for 2 h (2 h) and 2 h + 2 h of washout (2 + 2 h). The amounts of the total mRNA were normalized with the corresponding levels of GAPDH mRNA. The values are expressed as mean percentages ± S.E.M. with respect to the control (100%). * *p* < 0.05, ** *p* < 0.01, *** *p* < 0.001, Tukey’s multiple comparisons test, *n* = 3 independent samples. (**C**,**D**) Representative cropped Western blotting (upper panel) and densitometric analysis (lower panel) of HuD (**C**) and BDNF (**D**) proteins and the respective a-tubulin in the total homogenates of SH-SY5Y cells following exposure to solvent (PBS) or FA for 2 h and 2 + 2 h. Results are expressed as mean grey levels ratios (mean ± S.E.M.) of HuD/a-tubulin (**C**) and BDNF/a-tubulin (**D**) ×1000. * *p* < 0.05, ** *p* < 0.01, Tukey’s multiple comparisons test, *n* = 3–7 independent samples.

The obtained data indicate that a 2-h FA treatment is sufficient to trigger the HuD/BDNF cascade (Figure 5). Indeed, as expected, at this time, we observed an increase in the amount of both HuD and BDNF transcripts with no changes in the corresponding protein content. However, following 2 h of washout, we could detect a significant rise in both HuD and BDNF protein levels, thus mirroring the profile previously observed following 4 h of FA incubation. Following 2 h of washout, we also found a further increase in the mRNA levels of both HuD and BDNF in comparison to the corresponding amounts detected after 2 h of FA only.

**Figure 6:**
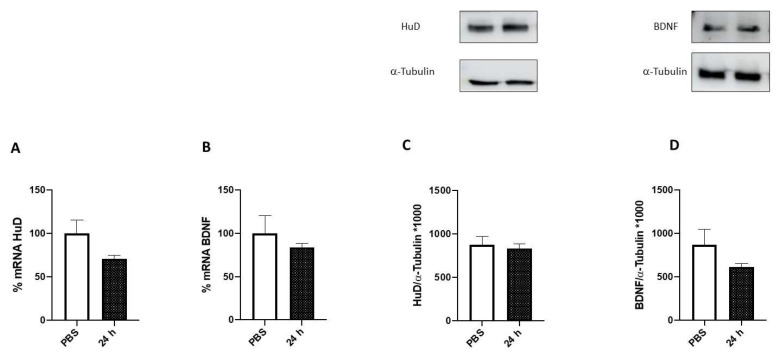
HuD and BDNF expression following 24 h of folic acid treatment. (**A**,**B**) Determination of HuD (**A**) and BDNF mRNA (**B**) levels via RT-PCR in SH-SY5Y cells exposed to solvent (phosphate-buffered saline; PBS) or folic acid (FA) for 24 h (24 h). The amounts of the total mRNA were normalized with the corresponding levels of GAPDH mRNA. The values are expressed as mean percentages ± S.E.M. with respect to the control (100%). (**C**,**D**) Representative cropped Western blotting (upper panel) and densitometric analysis (lower panel) of HuD (**C**) and BDNF (**D**) proteins and the respective a-tubulin in the total homogenates of SH-SY5Y cells following exposure to solvent (PBS) or FA for 24 h. Results are expressed as mean grey levels ratios (mean ± S.E.M.) of HuD/a-tubulin (**C**) and BDNF/a-tubulin (**D**) ×1000, *n* = 3–7 independent samples.

To further investigate the effects of a prolonged FA treatment on BDNF and HuD expression, cells were exposed to FA for 24 h. As shown in Figure 6, no changes were observed in HuD and BDNF expression either at the transcript or protein level.

### 2.4. HuD Post-Translational Modification after Prolonged Folic Acid Treatment

**Figure 7:**
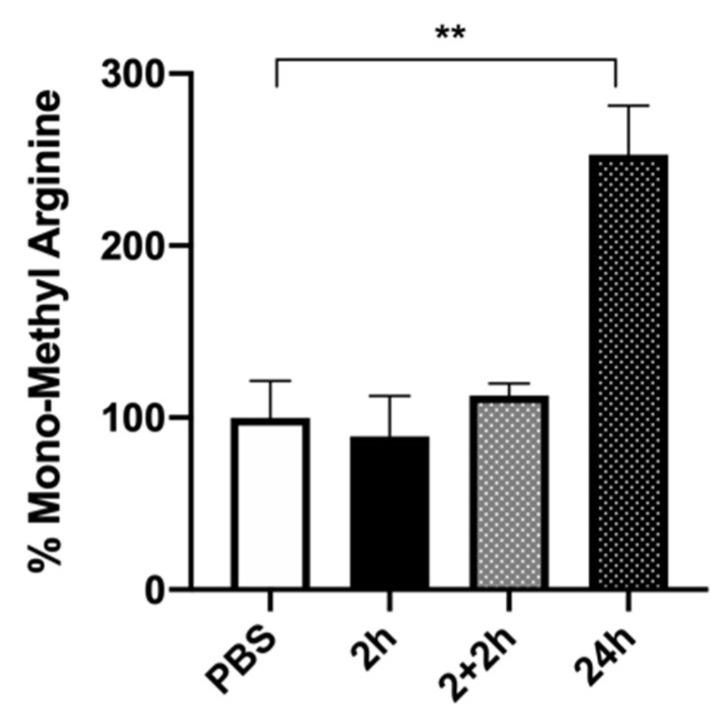
HuD methylation after folic acid treatment. Densitometric analysis of HuD methylation in arginine residues following folic acid exposure for 2 h (2 h), 2 h + 2 h of washout (2 + 2 h), or 24 h. The results are expressed as mean percentages + S.E.M. with respect to the control (PBS, 100%). ** *p* < 0.001, Dunnett’s multiple comparisons test, *n* = 3–6 independent samples. PBS: phosphate-buffered saline.

Lastly, to obtain further insight into the mechanism by which a prolonged FA exposure might modulate HuD function, and given that FA is a methyl donor, we performed immunoprecipitation experiments to investigate FA-mediated potential post-translational modifications in the HuD protein. In particular, we focused on the implication of arginine methylation, as this modification affects the activity of proteins involved in various cellular pathways, including nuclear-cytoplasmic signaling, transcriptional activation, and posttranscriptional modulation [24,35,39]. As reported in Figure 7, a significant increase in mono-methyl arginine after 24 h of FA exposure, compared to the treatment with PBS alone, was observed. Conversely, no HuD methylation was observed at 2 h of treatment or after washout (2 + 2 h), suggesting that FA specifically induces arginine methylation only after a longer timeframe. In our experimental conditions, this methylation likely affects HuD activity.

## 3. Discussion

RBPs are *trans*-acting factors involved in gene regulation at the post-transcriptional level. Through binding to target mRNA molecules, RBPs have a significant impact on their fate, primarily influencing their stability and/or translation [2,40,41]. One of the extensively studied RBPs is the ELAV family of proteins, also known as “Hu” proteins [42]. Among them, HuD activity has been extensively studied in neuronal development, plasticity, and regeneration. Further, in support of its key implication in the CNS, recent studies suggest that HuD misregulation might underlie neurological disorders, including neurodegenerative diseases such as Parkinson’s disease, Alzheimer’s disease, and amyotrophic lateral sclerosis. Thus, these discoveries emphasize the concept that HuD represents a promising focus for the creation of innovative pharmaceutical interventions intended to combat neurodegenerative disorders.

In this regard, in our previous study, we demonstrated that FA is a HuD binder [31]. Folic acid plays a crucial role in various aspects of CNS development, maintenance, and function. Substantial epidemiological evidence has been gathered on the role of folate in neural tube defects, neural development, and in counteracting neurodegenerative diseases. However, the specific mechanisms through which folate produces its effects have remained unclear, thus requiring further investigation. Based on our previous findings, in this study, we performed a deeper investigation into the effect of FA on HuD at the cellular level with possible future implications in the prevention of NDs. To this aim, the identification of the molecular targets whose expression and regulation is mediated by HuD, as well as a thorough understanding of their reciprocal interactions, was our first goal. Very recently, all the known transcripts controlled by HuD that play an important role in the CNS have been reviewed [43]. We processed the available data into a comprehensive PPI network (Figure 2). The analysis of this network revealed a remarkable degree of interconnections implicating the neuronal targets regulated by HuD. Among them, the BDNF stands out as the hub and bottleneck of the PPI network. Moreover, it is involved in 9 out of the 13 CNS-related pathological pathways examined (i.e., Huntington disease, amnestic disorder, toxic encephalopathy, cognitive disorder), and it is able to interact with an additional 14 targets, all under HuD control and also involved in CNS pathologies. In light of these considerations, we investigated the effects of FA on both the modulation of HuD and on the expression of the down-stream target BDNF.

Prior to conducting the experiments, we confirmed the non-toxic nature of FA through the assessment of mitochondrial activity, ensuring the safety of the concentrations used in the subsequent experiments performed in SHSY5Y neuronal cells. Notably, SH-SY5Y cells are commonly used as an in vitro model for studying neuronal function and disease. As depicted in Figure 4, the results demonstrated that FA exposure leads to a significant increase in both HuD and BDNF transcripts and proteins after 2 and 4 h of treatment, respectively. These data strongly indicate that HuD protein, once activated by FA, not only has a positive effect on its target transcript BDNF, but also acts on itself, thus boosting its own expression through a mechanism of auto-modulation.

Subsequently, we conducted additional experiments where the cells were exposed to folic acid for 2 h followed by a 2-h washout period (2 + 2 h; Figure 5) to assess whether this timeframe was sufficient to activate the HuD/BDNF cascade. Indeed, the results obtained in this experimental setting mirror, for both HuD and BDNF proteins, the profile previously observed following 4 h of FA incubation. Surprisingly, after 2 + 2 h treatment we found a further increase in the mRNA levels of both HuD and BDNF in comparison to the corresponding amounts detected after 2 h of FA only. This finding was unexpected when considering the results obtained following 4 h of FA treatment. In this regard, we may postulate that while a short exposure produces a long-lasting stimulation of the transcriptional phase, a prolonged incubation with FA (i.e., 4 h) can, instead, “freeze” the transcription process itself.

Moreover, the increase in HuD and BDNF expression was no longer detected after 24 h of folic acid exposure (Figure 6). This observation suggests the presence of a signaling shutdown mechanism occurring over time, probably acting on HuD functioning. To test this hypothesis, and given that FA is a methyl donor, which is a process requiring prolonged incubation times, we explored, via immunoprecipitation experiments, possible post-translational modifications in the HuD protein after 24 h of FA exposure. Intriguingly, we observed a methylation of HuD in arginine residues (Figure 7). As previously mentioned, the methylation of HuD in arginine residues has been shown to reduce HuD function and to negatively affect its activity on the down-stream targets [24]. Therefore, we can postulate that following 24 h of incubation, FA acts as an epigenetic modulator of HuD, switching off its activity.

Overall, the obtained results provide evidence for a time-dependent regulation of HuD mediated by folic acid. Namely, while a short-term exposure to FA leads to an increase in HuD activity, a long-term treatment induces a signaling shutdown, likely via methylation of HuD, with a consequent impact on the down-stream processes regulated by HuD, such as BDNF expression.

## 4. Material and Methods

### 4.1. Protein–Protein Interaction (PPI) Network

The Search Tool for Retrieval of Interacting Genes (STRING; https://string-db.org, accessed on 29 July 2022) database was exploited to build the PPI network among the CNS targets whose modulation is affected by HuD. Giving a list of proteins as an input, STRING can search for their neighbor interactors, namely the proteins that have direct interaction with the inputted proteins. Then, STRING can generate the PPI network consisting of all these proteins and all the interactions between them [43]. The interactions were analyzed by selecting “Homo sapiens” as organism and setting the confidence basis to 0.4 [44,45]. To evaluate the nodes in the PPI networks, it was imported in Cytoscape for the computing of several topological measures including degree (k) and between centrality (BC). The k and BC values are often used for detecting the hub or bottleneck in a network. The k of a node is defined as the number of edges linked to it. A node with a high k denotes a hub having many neighbors. The BC of a node is the proportion of the number of shortest paths passing through it to the number of all the shortest paths in the network, quantifying how often a node acts as a bridge along the shortest paths between two other nodes. A node with high BC has great influence on what flows in the network and has more control over the network. It can represent the bottleneck in the network. The protein nodes with high k or BC as the hubs or bottlenecks were considered.

### 4.2. Cell Culture

Human neuroblastoma SH-SY5Y cells were obtained from ATCC (Manassas, VA, USA) and cultured in a humidified incubator at 37 °C with 5% CO_2_. The SH-SY5Y cells were grown in Eagle’s minimum essential medium (EMEM) supplemented with 10% fetal bovine serum, 1% penicillin–streptomycin, L-glutamine (2 mM), nonessential amino acids (1 mM), and sodium pyruvate (1 mM).

### 4.3. MTT Assay

Mitochondrial enzymatic activity, as evaluation of the cell viability, was estimated via MTT [3-(4, 5-dimethylthiazol-2-yl)-2,5-diphenyltetrazolium bromide] assay (Sigma-Aldrich, Darmstadt, Germany). An amount of 200 mL of a suspension of 3.5 × 10^5^ cells/well was seeded into 96-well plates. The cells were treated with 100 nM, 1 μM, and 100 μM FA (solubilized in phosphate-buffered saline, PBS) for 24 and 48 h and then subjected to MTT assay following the protocol published in our previous paper [46]. The absorbance values were measured at 595 nm using a Synergy HT microplate reader (BioTek Instruments, Santa Clara, CA, USA), and the results were expressed as % with respect to control.

### 4.4. Western Blotting

SH-SY5Y cells were treated with 100 nM FA for 2, 4, and 24 h, and then they were homogenized in a specific cell lysis buffer (Cell Signaling Technology, Denvers, CO, USA). Proteins were diluted in 2X sodium dodecyl sulphate (SDS) protein gel loading solution, boiled for 5 min, separated on 12% SDS-polyacrylamide gel electrophoresis (SDS-PAGE), and processed following standard procedures [47]. The mouse monoclonal antibodies were diluted as follows: the anti-ELAVL4/HuD antibody (Sigma-Aldrich, Darmstadt, Germany ) at 1:1000; the anti-BDNF (anti-brain-derived neurotrophic factor; Sigma-Aldrich, Darmstadt, Germany ) at 1:500; and the anti-α-tubulin (Sigma-Aldrich, Darmstadt, Germany ) at 1:1000. The nitrocellulose membrane signals were detected via chemiluminescence (by using WesternBright^®^ ECL HRP substrate, Advansta, San Jose, CA, USA) by means of an Imager Amersham 680 detection system. All the experiments were performed in duplicate. Alpha-tubulin was used for data normalization. Statistical analysis was performed on the densitometric values obtained with the ImageJ image processing program.

### 4.5. Real-Time Quantitative RT-PCR

RNA was extracted from total homogenates using RNeasy Micro Plus Kit (Qiagen, Hilden, Germany). The reverse transcription was performed following standard procedures. PCR amplifications were carried out using the Rotor-Gene Q (Qiagen, Hilden, Germany) in the presence of QuantiTect SYBR Green PCR mix (Qiagen, Hilden, Germany) with primers predesigned by SIGMA. Primer sequences used were as follows: forward: 5′-CATCGTCAACTATTTACCCC-3′ and reverse: 5′-GTCCTGTAATTTTGTCTCTCAC-3′ (for ELAVL4/HuD); forward: 5′-AACCATAAGGACGCGGACTT-3′ and reverse: 5′-TGCAGTCTTTTTATCTGCCG-3′ (for BDNF); forward: 5′-CAGCAAGAGCACAAGAGGAAG-3′; and reverse: 5′-CAACTGTGAGGAGGGGAGATT-3′ (for GAPDH). The GAPDH mRNA was chosen as control since it is relatively stable during all the treatments, and it is unaffected by HuD because it does not possess ARE sequences (not shown). ELAVL4/HuD and BDNF mRNA expression was normalized over GAPDH mRNA.

### 4.6. Immunoprecipitation

Immunoprecipitation was performed according to a previously published protocol with minor modifications [48]. Immunoprecipitation was carried out at room temperature using 1 μg of an anti-HuD antibody (Sigma-Aldrich, Darmstadt, Germany ) per 50 μg of total proteins diluted in the immunoprecipitation buffer (50 mM Tris pH7.4, 150 mM NaCl, 1 mM MgCl_2_, 0.05% Igepal, 20 mM EDTA, 100 mM DTT, protease inhibitor cocktail, and RNAase inhibitor) in the presence of 50 μL A/G plus agarose (Santa Cruz Biotechnology Inc., Dallas, TX, USA). The sample, representing the immunoprecipitated HuD protein, was then subjected to Western blotting using an antibody recognizing methylated residues [Cell Signaling; Mono-Methyl Arginine (R*GG)]. An irrelevant antibody (Santa Cruz Biotechnology Inc., Dallas, TX, USA) with the same isotype as the specific immunoprecipitating antibody served as a negative control.

### 4.7. Statistical Analysis

The GraphPad Prism statistical package (version 9, San Diego, CA, USA) was used for the statistical analysis. The data were analyzed by analysis of variance (ANOVA) followed, when significant, by an appropriate post hoc comparison test, as detailed in the legends. Differences were considered statistically significant when *p*-value ≤ 0.05. The results are expressed as mean ± S.E.M.

## 5. Conclusions

In conclusion, our findings shed light on the potential neuroprotective effect of folic acid through the upregulation of HuD and BDNF expression. Of interest, considering that decreased levels of BDNF are associated with neurodegenerative diseases, including Alzheimer’s and Parkinson’s diseases, the observation that FA can increase BDNF expression holds promise for the prevention and treatment of these conditions. Furthermore, our findings also support HuD as a novel target of drugs focused on counteracting neurodegenerative diseases.

## Data Availability

The data presented in this study are available on request from the corresponding author.

## Supplementary Materials

The following supporting information can be downloaded at: https://www.mdpi.com/article/10.3390/ijms241512201/s1.
