# Supplementary material for: Short- and Long-Term Regulation of HuD: A Molecular Switch Mediated by Folic Acid?
Source: Int J Mol Sci. 2023 Jul 30;24(15):12201. doi: 10.3390/ijms241512201 (PMC10418318; doi:10.3390/ijms241512201)
Supplement: Supplementary file 1 [file ijms-24-12201-s001.zip › ijms-2502930-supplementary.pdf]

Supplementary Figure

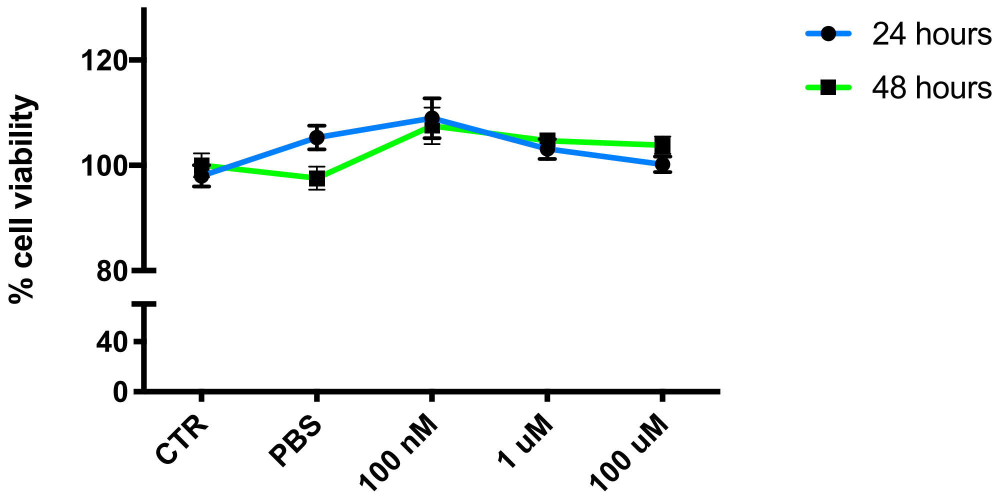

Figure S1: Effect of Folic Acid onSH-SY5Y mitochondrial activity. SH-SY5Y were exposed to different concentrations of folic acid for 24 hours and 48 hours. The results are expressed as mean + S.E.M. related to MTT measures as a percentage over controls (CTR, 100%). Data were analyzed by two-way ANOVA. PBS: phosphate-buffered saline.
